# Modeling the kinetics of interorgan arginine metabolism during bacterial sepsis in swine

**DOI:** 10.1152/ajpgi.00375.2024

**Published:** 2025-01-29

**Authors:** Caitlin Vonderohe, Douglas Burrin

**Affiliations:** USDA/ARS Children’s Nutrition Research Center, Section of Pediatric Gastroenterology, Hepatology and Nutrition, Department of Pediatrics, Baylor College of Medicine, Houston, Texas, United States

In this journal (1), Rice et al. use a unique, multicatheterized, swine model to assess the kinetics of arginine metabolism using stable isotopes in the early stages of sepsis. The study focuses on arginine metabolism because it is a critically important amino acid and is conditionally essential in neonates and in severe illness because the high arginine demand for protein, polyamines, and creatine synthesis exceeds its endogenous production (2). A key function of arginine is as a precursor for nitric oxide, which plays a central role in regulation of blood flow, tissue perfusion, and inflammation. In normal physiology, arginine requirements are met by dietary protein intake, but also by renal synthesis from citrulline produced almost exclusively by the intestine.

In their article, the clinical focus of Rice et al. (1) is sepsis, a life-threatening condition that occurs in response to an infection and causes widespread inflammation, tissue damage, organ failure, and potentially death. It can be triggered by various types of infections, including bacterial, viral, and fungal infections arising from the gut, lungs, urinary tract, or bloodstream. In the United States each year, over 2 million people develop sepsis leading to ~270,000 deaths and is the third leading cause of hospital deaths in the United States (3) The onset of sepsis induces a hypermetabolic state characterized by increased protein catabolism resulting in increased release of amino acids, including arginine, into circulation. Several studies have shown that sepsis is marked by an arginine-deficient state, despite the net release from protein catabolism, suggesting increased catabolism. Two key metabolic pathways for arginine catabolism are via nitric oxide synthase to produce citrulline and via arginase resulting in ornithine synthesis. Nitric oxide is critical for multiple cell functions, whereas ornithine is a precursor for synthesis of polyamines involved in cell proliferation (2).

This research group has a longstanding interest in arginine metabolism and its role in the pathogenesis and potential therapies for treatment of sepsis (4–6). In this current report, they have used a recently developed bacterial sepsis approach using infusion of a live clinical isolate of *Pseudomonas aeruginosa* (7) using multicatheterized swine combined with stable isotopes of various amino acids, including arginine. *Pseudomonas aeruginosa* is a gram-negative bacteria that is often isolated from patients with sepsis and also provides a broad physiological inflammatory stimulus beyond that of specific toxins such as lipopolysaccharide (8). This approach has advantages because swine have similar physiology and metabolism to humans, and measurements of transorgan balance enable quantification of whole-body exchange of arginine and other amino acids that are ethically difficult to quantify in humans, especially those with critical illness like sepsis.

A main finding from the study was the large net release of amino acids from the hindquarters, comprised mostly of skeletal muscle and not the intestine, in response to sepsis, reflecting the large protein catabolic effect. The net hindquarter release of arginine was sixfold greater in septic versus control conditions. Interestingly, this large release of arginine into the circulation, triggered by sepsis, did not result in an increase in circulating arterial arginine concentrations, nor was there a decrease in plasma arginine as has been reported frequently in the past (4). This implies that the decrease in circulating arginine level previously observed in patients with sepsis and animal models in sepsis or endotoxemia may occur later in the process of adaptation to infection (4). The large hindquarter release of arginine and other amino acids during sepsis resulted in a commensurate increase in whole body urea production, and this was reflected in the net splanchnic (reflecting liver and gastrointestinal tissue) and liver uptake of arginine in this pig model. A major metabolic fate of arginine is the conversion to ornithine via arginase, which is expressed in multiple cell types, including hepatocytes and immune cells such as macrophages, and is activated during the inflammatory response (2). The calculated rates of whole-body arginine to ornithine and arginine to urea conversion were markedly higher with sepsis and support this massive metabolism of arginine via arginase activity. In contrast, there was not a significant increase in whole-body nitric oxide (NO) production. The latter is unexpected as a large pro-inflammatory stimulus like sepsis would typically increase NO production even though it represents a small fraction of overall arginine metabolism in the body, usually 5%–10% (9, 10).

Finally, another informative feature of this report is the use of covariate analysis (ANCOVA) of the data to account for sources of variance in the arterial-venous difference measurements. The transorgan balance approach often combines small arterial-venous differences in analyte concentrations (i.e., arginine) and these get amplified when combined with variable rates of blood flow. The authors show in supplemental results that the ANCOVA model was useful to reduce variance and improved the sensitivity to determine statistical difference and provide some caveats as to how to handle data using these analyses.

In summary, this study highlights the complexity of preclinical studies to assess the metabolic impact of sepsis. There has been debate in the field as to what is the best approach to simulate sepsis in animal models, some using endotoxin, cecal-ligation and puncture, where others infuse various species of live bacteria (11). An important goal in refining future preclinical models of sepsis also should be increasing the standardization and reporting of details of the experimental design, approach, and outcomes used (12). This study demonstrates how the use of transorgan amino acid kinetics using stable isotopes can be combined with clinically-relevant bacterial sepsis in swine to assess the longitudinal changes in metabolism. These features highlight the advantages of using swine and hopefully with spur other groups to examine more detailed changes in tissue and organ specific immune responses to infection as well as how therapeutic interventions can stem the pro-inflammatory response and tissue injury.
